# Perceived corporate social responsibility, ethical leadership, and moral reflectiveness impact on pro-environmental behavior among employees of small and medium enterprises: A double-mediation model

**DOI:** 10.3389/fpsyg.2022.967859

**Published:** 2022-11-23

**Authors:** Mourad Mansour, Nida Aman, Basheer M. Al-Ghazali, Syed Haider Ali Shah

**Affiliations:** ^1^Department of Management and Marketing, King Fahd University of Petroleum and Minerals, Dhahran, Saudi Arabia; ^2^Center for Finance and Digital Economy, King Fahd University of Petroleum and Minerals, Dhahran, Saudi Arabia; ^3^Management Sciences Department, Bahria University, Islamabad, Pakistan; ^4^Department of Business Administration-DCC, King Fahd University and Minerals, Dhahran, Saudi Arabia; ^5^Business Studies Department, Bahria University, Islamabad, Pakistan

**Keywords:** CSR, ethical leadership, moral reflectiveness, employees’ pro-environmental behavior, SME

## Abstract

Corporate social responsibility (CSR) is ever-evolving concept and gaining significance in the business world. This research proposes a research model that elucidates the mechanism by which Corporate social responsibility (CSR), ethical leadership, and moral reflectiveness promote pro-environmental behavior among employees of Small and Medium Enterprises (SMEs). In today’s ever-changing environment, small and medium companies (SMEs) are critical for any economy to thrive and prosper. SMEs account for more than 30% of the GDP in Pakistan. However, little is known about the processes by which type of leadership influence the pro-environmental behavior, or the circumstances under which such impacts are strengthened or mitigated. The current study attempted to fill the gap by investigating a dual process model in which ethical leadership and a moral reflectiveness acted as two explanatory mechanisms in the impact of Corporate social responsibility (CSR) on pro-environmental behavior among employees working in small and medium enterprises (SMEs). The structural equation modeling technique was used to test the research model’s hypothesized relationships. A survey was used to collect data from 390 employees. Results showed that perceived CSR directly impacted moral reflectiveness and ethical leadership. Moreover, the moral reflectiveness and ethical leadership mediated the relationship between the perceived CSR and pro-environmental behavior. These findings contributed significantly to perceived CSR, ethical leadership, moral reflectiveness, and pro-environmental behavior among employees by exploring and integrating the holistic research work into one framework to add to the body of knowledge. Practical implications and future research directions are also highlighted.

## Introduction

Environmental considerations put a lot of pressure on businesses to become more environmentally beneficial (Park et al., 2012; Afsar et al., 2020). Green policies and practices are being adopted by an increasing number of organizations in order to improve economic benefits and environmental performance (Ardito and Dangelico, 2018). Organizational environmental success, however, is dependent not just on strict rules and regulations, but also on employees’ positive response to various environmental issues through pro-environmental behavior (Kim et al., 2017; Unsworth and McNeill, 2017). Employees’ pro-environmental behavior is critical to a company’s environmental management success since it improves the company’s overall environmental performance (Lu et al., 2017; Wesselink et al., 2017; Afsar et al., 2018). Employee’s pro-environmental behaviors refer to “discretionary employee actions that contribute to the environmental sustainability of the employer organization but are not under the control of any formal environmental management policies or system” (Kim et al., 2017, p. 1337). Recycling and reusing, discovering sustainable ways of working, and generating and implementing ideas for decreasing the company’s environmental impact are all examples of pro-environmental practices Graves et al. (2013). The question of how to make it easier for employees to engage in pro-environmental conduct has gotten a lot of attention in recent years. Importantly, researchers have concentrated on determining the factors that influence them.

Corporate social responsibility has been highlighted as an important antecedent in the literature. An organization’s CSR-specific initiatives are undertaken in order to gain the belief of all stakeholders that such actions would result in positive outcomes for all stakeholders (Afridi et al., 2020). Employees are critical stakeholders in an organization’s success (Molnár et al., 2021). There is a common misperception in CSR discussions that CSR initiatives are only carried out by large, profitable businesses because they can afford and have the resources for CSR (Jansson et al., 2017; Meyer et al., 2017; Lucky, 2018; Bikefe et al., 2020). However, in the global CSR environment, Small and Medium Enterprises (SMEs) also play an important role as they also carry out the CSR practices which have the positive and significant impact on the organization and society as well (Jansson et al., 2017; López-Pérez et al., 2017; Bikefe et al., 2020). Corporate social responsibility is a way for businesses, including SMEs, to contribute to societal causes (Meyer et al., 2017; Schmidt et al., 2018; Yeo and Carter, 2018). Enterprises can utilize CSR to help reduce the negative effects their activities may have on the environment, as businesses contribute to environmental depletion (Jamali et al., 2017; Doshmanli et al., 2018; Melissen et al., 2018). Corporate social responsibility is implemented through good community relations, as well as optimum labor, manufacturing, and environmental policies (Van Woerkom and Zeijlrozema, 2017; Bikefe et al., 2020). In an organizational context, since employees spend a considerable amount of their daily time at work, influencing their environmental behavior can aid in reducing the organization’s overall environmental impact. Well-established companies invest a large amount of money in various CSR initiatives (Lii and Lee, 2012). In addition to that, the effectiveness and usefulness of a CSR plan are dependent on employee participation, as people play a critical strategic role in achieving various organizational goals (Wu et al., 2021). This research agrees with Carroll, the field’s founder, when it comes to defining CSR as “the economic, legal, ethical and philanthropic responsibility of an enterprise toward different stakeholders” (Carroll, 1979, 2016 16). It is critical to involve an organization’s employees in various CSR-related activities because without active participation from internal stakeholders, the organization’s CSR efforts will fail. Further, Expectations of a corporation achieving sustainability goals will not be achieved until the internal stakeholders are included in the process (Kucharska and Kowalczyk, 2019). Internal stakeholders (employees) are crucial for an organization’s execution of CSR-specific programs, according to different researchers (Tian and Robertson, 2019; Schaefer et al., 2020).

Moreover, this research study uses Brown et al.’s (2005) concept of EL when defining EL which is “EL refers to a leadership style which is characterized by respect for ethical norms, values, and dignity for the employees through interactive communication and involving them into organizational decision making.” The role of Ethical leadership (EL) has also been shown to have a positive impact on employee behavior by researchers (Afsar et al., 2020; Maqsoom et al., 2020). However, it is unclear how ethical leaders might impact their followers’ CSR-related actions, such as employee pro-environmental behavior (EB; Wu et al., 2021). According to Lawton and Páez (2015) Ethical leadership is defined as “it involves some aspect of personal conduct, deemed ethically appropriate, in decision-making and developing relations with others, such that these others are inspired to follow.” Ethical leaders believe in normatively adequate ethical behavior and express these behaviors through their own activities. Such leaders aim to boost their employees’ hopes and convert their self-concepts and personal standards to a higher level of goals and demands (Brown et al., 2005). Whereas the same is advocated by Kollmuss and Agyeman (2002) to define EL “it is the behavior of employees to minimize their negative impact on the natural and built world” (p. 243). Some researchers have emphasized the relevance of leadership in PEB adoption (Khan et al., 2019; Ren et al., 2020). Ethical leaders involve in building the vision which incorporates the various initiatives leading toward the environment and particularly the employee’s behavior toward pro-environmental concerns and encourage them exhibiting the pro-environmental behavior (Shah et al., 2021a; Wu et al., 2021; Al-Ghazali et al., 2022). Ethical leaders are always concerned for the organizational pro-environmental behavior (Molnár et al., 2021). Similarly, the despite of being so important the role of perceived CSR and ethical leadership still there are few studies which investigated the perceived CSR and ethical leadership impact on pro-environmental behavior particularly in the developing country like Pakistan (Shah et al., 2021b; Al-Ghazali et al., 2022; Sun et al., 2022). There are mixed findings of those investigations, some studies found a significant relationship and some studies found insignificant relationships, so that is why we mentioned that additional research is required to find out in developing country context, particularly in SMEs. As a result, the main objective of this study is to investigate the impact of employees’ CSR perceptions on PEB, while suggesting that EL mediates this relationship.

With the same notion, the association between perceived CSR and employee work results has been found to be influenced by individual characteristics and personality features (Rupp et al., 2013). Individual differences in the degree of morally guided reflection people engage in about their everyday experiences and the extent to which an individual contemplates moral matters in their daily encounters and decisions are defined as moral reflectiveness (Reynolds, 2008). Individuals who engage in pro-environmental activity at work do not expect financial rewards to flow directly to them (Afsar and Umrani, 2020). Saving energy by turning off lights or taking the stairs instead of the elevator, for example, would not benefit people on a personal level because organizations would gain the true economic advantage, if an employee does not engage in pro-environmental conduct, negative consequences do not immediately affect him or her (Afsar and Umrani, 2020). While groups, organizations, and society, may suffer more and the organization should consider the PEB of employees. As a result, it is a person’s moral obligation to engage in pro-environmental acts that can inspire them. Perceived CSR drives the moral reflection in employees which, in turn, leads to pro-environmental behavior of employees.

In addition to that, currently, the environmental concerns and issues are highlighted at the government level of Pakistan and now the government is taking initiatives to address these challenges and threat to environment (Khan et al., 2020; Ahmad et al., 2021). In this study, the Punjab province of Pakistan has been selected because majority of the SMEs are operating there (Von Weltzien Hoivik, 2011; Small and Medium Enterprises Development Authority, 2020; Shah et al., 2021b; Sun et al., 2022). Moreover, perceived CSR and ethical leadership can trigger the pro-environmental behavior in employees of SMEs particularly because of the positive influence of the ethical leadership (Wu et al., 2021; Hongxin et al., 2022). Various studies emphasized on transformational leadership on pro-environmental behavior (Peng et al., 2021; Deng et al., 2022). However, the role of perceived CSR and ethical leadership as a mediating variable is less investigated in developing country like Pakistan, particularly in SME sector (Shah et al., 2021a). Moreover, the size and profile of SMEs in the region is further discussed in methodology and analysis section. In addition to that, very few studies have been conducted to investigate the mediating role of Moral Reflectiveness and between the perceived CSR and pro-environmental behavior.

**Figure 1:**
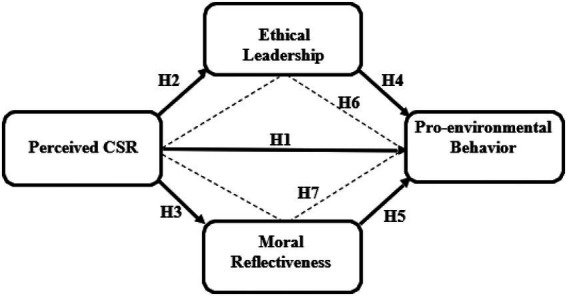
Presents the conceptual framework of this study. * Straight lines show the direct effects. ** Dotted lines show the indirect effects.

This paper fills several current gaps in the literature. First, it develops and tests a model in which moral reflectiveness and EL, serve as mediating factors in order to investigate the impact of perceived CSR on employee pro-environmental behavior in SMEs. Hence, this study extends research on the mediating role of moral reflectiveness on employee pro-environmental behavior. This research work is aimed into the mediating influence of an employee’s moral reflectiveness on the relationship between CSR and pro-environmental behavior, in order to gain a better understanding of the social psychological processes that enable people to engage in pro-environmental activities at work (Takeuchi et al., 2007; Babiak and Trendafilova, 2011; Zientara and Zamojska, 2018; Afsar and Umrani, 2020). Leadership has been highlighted as an important antecedent in this literature because leaders, as organizational agents, have a profound effect on employees in their treatment. However, limited research has focused into whether and how ethical leadership may encourage pro-environmental behavior among employees through CSR. As CSR is a driver that influences employee behavior to reduce environmental negative effects in SMEs of Pakistan. Although a number of studies have explored into the impact of CSR activities on employee behavior and outcomes, the majority of them have focused on the direct link between CSR and employee outcomes. Another gap this study fills is to investigate the impact of CSR though indirect effect of EL on PEB in SME sector of Pakistan. The current study explores all the relationships in Pakistan’s SME sector, which is purposefully chosen by the researchers because Pakistan’s SME sector is a major sector where an organized CSR effort is evident. However, the majority of these efforts have focused on the charitable aspect of CSR (Al-Gasawneh et al., 2021), ignoring the environmental considerations covered by CSR (Szegedi et al., 2020). As a result, the study extends the existing CSR literature by providing empirical evidence on its positive outcomes from a developing country context, that is, Pakistan. The proposed model of the current study is presented in Figure 1.

## Literature review, theoretical foundation, and hypotheses development

### This study framework is based on ability motivation opportunity because of the several reasons, which are discussed in below paragraphs

According to the AMO theory, the three key components are crucial in order to form the HPWS. These three aspects are ability, motivation, and opportunity (AMO; Appelbaum et al., 2000). The ability to develop and use a variety of techniques to carry out a range of activities and obligations with pertinent knowledge and abilities; examples of these techniques include training and development and recruiting and selection programs.

In the same manner, goals can be achieved when workers are encouraged through appropriate financial, non-financial, and financial performance management (FPM) which refers to way of managing the financial results across the organization and monitoring the whole process, whereas non-financial performance measurement indicates deficiencies in those areas that can affect the long-term strategic success of an organization (Sun et al., 2022). Lastly, the opportunity represents the organization’s policies, which are embedded with the ideas of personal liberty, involvement in decision-making, and the free flow of information with workers. In addition to that, Building employee green training capabilities and getting suppliers’ and customers’ cooperation are essential for promoting the PEB (Yu et al., 2020; Ding et al., 2022; Sun et al., 2022).

Moreover, the relationship between the Perception of CSR, Ethical leadership, and employees is a crucial resource for the company, and the primary objective of the green human resource management department is to develop and provide opportunities for growth in addition to the incentives and rewards that could boost their competitive advantage and raise performance to the next level (Boxall and Steeneveld, 1999; Sun et al., 2022). Hence, this study foundation is based on the organization’s structure and procedures which are closely related to its human capital development, which further strengthen the implementation on firm’s policies and practices which result in better organizational outcomes that allow it to outperform competitors (Singh et al., 2020; Khan et al., 2021; Sun et al., 2022).

The AMO theory is an essential theoretical paradigm in human resource management (Boxall et al., 2007; Sidney et al., 2022). The significance of employees’ abilities, opportunities, and motivations in influencing employee’s performance is emphasized by the AMO theory. This integrative perception reveals how and why top-management strategy and HRM practices support company performance.

Based on AMO theory, it highlights the previous research studies suggested that CSR and EL promote the green employee behavior (Sidney et al., 2022) through different mechanisms of social and psychological factors like citizenship behavior with respect to environment which consequently impact the employee’s green behavior and green innovation (Pham et al., 2019; Pinzone et al., 2019; Hameed et al., 2020; Singh et al., 2020). Previous research studies have shown the link of CSR on PEB and EL on PEB. Furthermore, elaborated that CSR and EL contribute in promoting the pro-environmental behavior (Pham et al., 2019; Pinzone et al., 2019; Hameed et al., 2021; Sidney et al., 2022). Based on this, this study develops the argument that CSR practices along with the EL aiming to attract, motivate and value the employee’s behavior toward environmentally friendly goals and may strengthen their perception toward organization which ultimately result in PEB. Moreover, the CSR and EL practices provide the support and organizational environment in which the employee’s care for sustainability and that reflects in the behavior of employees toward environment (Boselie et al., 2005). AMO further strengthens the employee’s attachment with work environment (Sidney et al., 2022). The main explanation is that AMO components affect the employee’s behavior and their valuable environmental ideas which are unique and combined at individual and situational elements promoted through CSR and EL (Jia et al., 2018; Sidney et al., 2022). Hence, this study investigates CSR and EL impact on PEB using AMO theory because of its close association with green human resource management.

### Pro-environmental behavior

According to Ramus and Killmer (2007) pro-environmental behavior has three dimensions. The first dimension is the prosocial nature of pro-environmental acts that benefit both individuals and organizations at the same time. The second component is discretionary nature, where employees engage in pro-environmental activities that are not required, such as turning off lights and taking the stairs rather than the elevator. The third component is the extra role nature of pro-environmental behavior, it states that environmental efforts in improving an organization’s environmental performance and protection of it are not explicitly included in job descriptions, employees do so for the organization’s green image. The definition of pro-environmental in this study is based on Kollmuss and Agyeman's (2002) definition. Which states that “behavior that consciously seeks to minimize the negative impact of one’s actions on the natural and built world.” Various research studies conducted on Individual level variables such as personal qualities, self-efficacy, environmental values, motivation, biological and ecological concerns, and habit, general awareness of nature, and environmental knowledge have been found to be major antecedents of an employee’s pro-environmental behavior (Sarkar, 2008; Afsar and Umrani, 2020), as well as some organizational-level variables including top-management support, green HRM practices, company culture, and corporate environmentalism (Boiral and Paillé, 2012). McDonald (2014) thoroughly reviewed frameworks from general pro-environmental and workplace pro-environmental behavior literature to construct a conceptual framework of pro-environmental behavior in the workplace. He reviewed previous research on general pro-environmental behaviors, finding that environmental concern, personal norms, habits, self-efficacy, stress, environmental awareness, Stress, knowledge, social structure (traditions, moral codes, and institutions), and social identity all have an impact. Similarly, a number of recent research have looked into the challenges of developing and revising frameworks to explain employees’ pro-environmental work behavior. Surprisingly, though, the majority of these researches were conceptual in essence (Lo et al., 2012; Lülfs and Hahn, 2014; McDonald, 2014; Norton et al., 2015). There are just a few empirical investigations on the elements that influence employee pro-environmental behavior.

### Corporate social responsibility and pro-environmental behavior

Corporate social responsibility is defined as an organization’s activities that go beyond governmental legislation to improve society (Gatti et al., 2019). Those businesses that are taking the CSR initiatives are considered to gain the benefits in multiple ways such as improved performance (Cho et al., 2019), minimizing the cost (Moggi et al., 2018), brand loyalty (Aljarah and Ibrahim, 2020), and ultimately gain the status of positive brand image (Ramesh et al., 2019). Corporate social responsibility is defined as an organization’s operations that benefit society. Employees’ positive actions are triggered when a firm engages in CSR initiatives that include environmental and social responsibilities which is CSR (Baughn et al., 2007; Afsar et al., 2018; Deng et al., 2022; Hongxin et al., 2022). According to Arnaud and Sekerka (2010), firms that are socially responsible share their CSR practices with their workforce through e-mails, newsletters, seminars, and other means. As a result, employees have a better understanding of contemporary social and environmental challenges, as well as how their companies are helping to protect the environment and society. Employees’ desire to establish and implement Eco initiatives is favorably correlated with a supportive work environment characterized by CSR activities (Babiak and Trendafilova, 2011; Afsar et al., 2020). In addition to that, employees who learn and share environmental principles are more likely to align their own values with the organization’s pro-environmental values. This creates an environment in which employees are more likely to engage in sustainable practices (Vallaster, 2017). Corporate social responsibility activities have been shown to improve emotional, behavioral, and attitude results in the workplace (Afsar et al., 2018). Employees who see their firms as more socially responsible are more likely to engage in Organizational Citizenship Behavior, according to Gkorezis and Petridou (2017). Pro-environmental behavior is one type of OCB that is particularly relevant. Hence, based on the above arguments, the following hypothesize has been developed:

*H1*: Perceived corporate social responsibility is positively associated with an employee's pro-environmental behavior.

### Corporate social responsibility, ethical leadership, and pro-environmental behavior

Beyond stakeholder management, the development of leaders who can promote ethical value within an organization is an important part of a socially responsible organization (Zhu et al., 2014). Research studies elaborated that the genuine tone for sustainability and long-term productivity for all stakeholders, including employees, is expected to be established by an organizational culture characterized by CSR and ethical leadership Lin and Liu (2017). A socially responsible company maintains good relations (including leaders) with care and consideration, fostering a good attitude in the workforce (Supanti et al., 2015). Moreover, in a socially responsible organization, corporate leaders are recognized as valuable resources. This esteemed treatment of corporate leaders transforms them into happy employees who readily engage in ethical codes of conduct while dealing with their employees in the workplace (Pasricha et al., 2018; Budur and Demir, 2019; Dey et al., 2022;Martin et al., 2022). As a result, a socially responsible organization’s trademark is effectively involving people in accomplishing company objectives, including sustainability Glavas (2016). Various studies have stated that CSR and ethical leadership are closely linked. To sum up, by applying ethical ideals and sustainable work practices in a firm, CSR as a business system may provide actual profits for all stakeholders (Sial et al., 2018; Chen et al., 2020; Molnár et al., 2021). This concept of a socially responsible corporation that works in the greater good of society and the environment is passed down to corporate leaders, who in turn learn to act ethically (Wu et al., 2021). As a result, corporate leaders are supposed to follow ethical guidelines. Moreover, an ethical leader is required to promote ethical behavior in the organization, and followers are expected to learn this ethical behavior. In this regard, the authors suggest that ethical leaders not only engage in ethical activities themselves, but also transmit to their followers the value of such ethical behavior for the greater good of many stakeholders, including the community and the environment (Moore et al., 2019; Wu et al., 2021). Employees’ extra-role performance and environmental behavior are stimulated in the presence of an ethical leader, according to various research studies (Ahmad et al., 2019; Saleem et al., 2020).

Additionally, previous research has shown that the ethical conduct of company leaders can influence employees’ perceptions of CSR-related activities (De Roeck and Farooq, 2018; Mostafa and Shen, 2019; Nejati et al., 2019). Employee participation in CSR-related activities can be increased by selecting an appropriate leadership style within a firm this paper reference. Corporate leaders play a critical role in involving their workforce in various CSR endeavors (Edinger-Schons et al., 2019). Furthermore, the values and attitudes of leaders who promote CSR practices have an impact on followers’ participation in CSR-related activities. Enterprises need ethical leaders who can encourage followers to engage in extra-role or voluntary actions in order to achieve CSR-specific results. Ethical leaders are intended to inspire their followers’ ethical behavior, assisting them in becoming ethical individuals who contribute positively to the preservation of society and nature (Engelbrecht et al., 2017). As a result, ethical leaders may encourage their employees to engage in CSR efforts. Ethical leaders are critical for increasing ethical ideals because they urge firms to be more socially and ecologically concerned. An ethical leader could be CSR-oriented, modeling, and encouraging followers to practice environmental protection (Ferreira, 2017).

Hence, based on the above arguments, the following hypothesize has been developed

*Hypothesis 2*: Perceived corporate social responsibility is positively associated with ethical leadership.

*Hypothesis 4*: Ethical leadership is positively associated with pro-environmental behavior.

*Hypothesis 6*: Ethical leadership mediates the relationship between PCSR and employees’ pro-environmental behavior.

### Corporate social responsibility, pro-environmental behavior, and moral reflectiveness

The association between perceived CSR and employee work results has been found to be influenced by individual characteristics and personality features (Rupp et al., 2013). Individual personality features vary, and as a result, people engage in a variety of actions *via* underlying motivational systems (Wong et al., 2008). Employee behavior at work is affected by personality features because they have a higher influence on behavior. For instance, employees’ green workplace behavior has been proven to be influenced by conscientiousness (Kim et al., 2017; Afsar and Umrani, 2020; Shah et al., 2021a). This study uses this assertion to consider moral reflectiveness as a proximal purpose of pro-environmental behavior. People’s experiences are generally structured by their underlying moral beliefs (Davison et al., 2009). These beliefs provide a framework for making decisions, interpreting events, and acting on them. According to Rupp et al. (2013), the impact of CSR on OCB is stronger for employees who sustain a high level of moral identity. Individual morality motivates individual interest and commitment to environmental issues, making moral reflectiveness a proximal motivating factor of pro-environmental conduct (Feinberg and Willer, 2013). Employees who are morally reflective are more likely to care about morality, and their moral systems and moral beliefs are internalized, resulting in favorable workplace behavior (Afsar and Umrani, 2020). Employees’ feelings of meaningful existence can be strengthened, according to Folger et al. (2005), if the firm supports and implements CSR initiatives. When employees believe their companies are socially responsible, this significance grows even more. Organizations must encourage employees to participate in CSR activities without giving the idea that CSR is solely for the purpose of satisfying stakeholders or avoiding lawsuits, but that all CSR actions are meant to benefit society and the environment, assuring long-term development (Folger et al., 2005). Employees then regard such CSR initiatives as ethical and morally correct (Aguinis and Glavas, 2017). The desire to do what is ethically good characterizes actions for employees with moral reflectiveness (Beugré, 2009). Extending this reasoning, moral reflectiveness would lead to pro-environmental behavior because, even if an individual receives no monetary advantage or other monetary benefits, doing what is ethically and morally good is meaningful since it aligns with one’s own values. According to Rupp et al., 2013, employees’ moral identities are influenced by their perceptions of CSR. People who really care about moral issues respect the welfare of others, according to Kim et al. (2017), and pro-environmental behavior may be a means to fulfill their moral concerns. When a person believes that his or her organization is responsible to society and the environment, he or she will engage in prosocial and environmental activities, but only if his or her own moral motives, moral schemas, and moral ideals lead them to do so (Afsar and Umrani, 2020). Hence, based on the above arguments, the following hypothesize have been developed

*Hypothesis 3*: Perceived corporate social responsibility is positively associated with moral reflectiveness.

*Hypothesis 5*: Moral reflectiveness has a positive association with pro-environmental behavior.

*Hypothesis 7*: Moral reflectiveness mediates the effect of perceived corporate social responsibility on pro-environmental behavior.

## Conceptual framework of the study

The Conceptual framework of the current study is presented in Figure 1

## Materials and methods

### Participants and procedure

The data was gathered from middle-level managers at small and medium-sized enterprises (SMEs). In Pakistan’s Punjab province, more than 66 percent of SMEs are operating. According to Small and Medium Enterprises Development Authority (2020), there were 15,833 SMEs registered in Punjab. A total of 360 companies were chosen from six categories (Textile, Leather/Footwear, Sports, Food and Beverages, Metal, and Wood and Furniture). This study used a cluster sampling technique to collect data from 390 middle-level managers (Bhutta et al., 2009). The six industries listed above were grouped together to form clusters and 650 questionnaires were distributed based on their proportion of the total population. Tables 1, 2 present industry profile and questionnaires distribution and collection.

**Table 1:** Industry profile.

Sr. no.	Industry	%	Firms
1	Textile	16.66	60
2	Leather/Footwear	16.94	61
3	Sports	15.55	56
4	Food and beverages	16.11	58
5	Metal	18.05	65
6	Wood and furniture	16.66	60
Total		100	360

**Table 2:** Questionnaires distributed and collected.

Cluster	Questionnaires distributed	Questionnaires received
Lahore	150	110
Multan	130	88
Sialkot	110	82
Gujrat	100	75
Faisalabad	90	67
Gujranwala	70	58
Total	650	480

### Data collection instruments

To assess perceived CSR, this study adapted a scale from Turker (2009). Items for the pro-environmental behavior scale were taken from Robertson and Barling (2013). There were a total of 12 items used to assess pro-environmental behavior. Some examples are: At work, I take stairs instead of elevators to save energy” and “I perform tasks that are expected of me in environmentally friendly ways.” Moreover, A five-item scale established by Reynolds (2008) was used to assess moral reflectiveness. Sample items are “I regularly think about the ethical implications of my decisions” and “I often reflect on the moral aspects of my decisions.” In order to measure the ethical leadership, this study employed the 10 item scale of Brown et al. (2005).

## Analysis and results

Data must first be prepared for analysis before it can be analyzed. To achieve a normal distribution of data, The initial stage is to identify and solve the missing values. The imputation method was developed for this reason and the mean substitution technique was applied within the scope of this method (Hair et al., 2014). Furthermore, to determine the extreme values, the Mahalanobis D2 value was used. In this research, there were no extreme values identified. Additionally, kurtosis and skewness metrics were used to determine whether the data set followed a normal distribution. The data set had a normal distribution because the kurtosis and skewness values were 2.512 and 1.374, respectively (Kline, 2011). Common method biases were carried out through Harman single-factor test. In this research, it was discovered that in the absence of factor rotation, the characteristic root of the common factor with the greatest explanatory power is 11.258, which explains 40.145 of the total variation. There is no single factor that accounts for the majority of covariance between independent and dependent variables. It demonstrates that there is no significant common method bias in this research.

The means, standard deviations, correlations, and reliability statistics (Cronbach’s alphas) are presented in Table 3, while the profile of the respondents is shown in Table 4. The correlations between the predictor, mediators, and outcome were significantly positively associated, as shown in the table, providing preliminary support for our hypotheses, while Table 4 represents the profile of the respondents.

**Table 3:** Mean, standard deviation, and intercorrelations.

**S. no**	**All variables**	**Mean (SD)**	**1**	**2**	**3**	**4**
1	Perceived CSR	3.65 (0.81)	**(0.851)**			
2	Ethical leadership	3.33 (0.63)	0.234^*^	**(0.867)**		
3	Moral reflectiveness	3.88 (0.94)	0.551^**^	0.145^**^	**(0.840)**	
4	Pro-environmental behaviors	3.23 (0.87)	0.666^*^	0.455^**^	0.563^**^	**(0.936)**

** Correlation is significant at the 0.01 level (2-tailed).

* Correlation is significant at the 0.05 level (2-tailed). Values in bold are the Cronbach alphas. SD Standard Deviation.

**Table 4:** Profile of the respondents.

Demographics	No. of respondents	Percentage (%)
Gender		
Male	330	75
Female	110	25
Age		
<40 years	280	64
More than 40 years	160	36
Education		
Bachelors	380	86
Masters	60	14
Experience		
<5 years	139	31
5–10 years	191	43
>10 years	110	25

### Measurement model

Convergent and discriminant validity were investigated using a series of confirmatory factor analyses (CFAs). SPSS AMOS 24 was used to determine the goodness of fit for all variables. The four-factor model (perceived CSR, Ethical leadership, Moral reflectiveness, and pro-environmental behavior) was found to be a better fit to the data, when compared to the three-factor, two-factor, and one-factor models, where all the components loaded on a single factor (see Table 5). At the 0.001 level, all of the factor loadings were determined to be significant, indicating convergent validity (Gerbing and Anderson, 1988; see Table 6) Furthermore, we used the Cronbach’s alpha for all constructs, which were found to be above to validate the excellent reliability. 70 (see Table 6). The average variance extracted (AVE) values were higher than 0.50, as shown in Tables 6, 7. Furthermore, composite reliability values exceeded AVE values. This further supports the suggested model’s convergent validity (Hair et al., 2010). Comparing the average shared variance and maximum shared variance values, which were found to be less than their respective AVE values, the discriminant validity was determined (see Table 7). As a result, construct validity for all constructs was established. The variance inflation factor was used to check for multicollinearity, and it ranged from 1.54 to 4.93 (<10; O’brien, 2007) indicating that there were no difficulties with multicollinearity.

**Table 5:** Results of model comparisons using a CFA approach.

Model	λ2	df	TLI	CFI	IFI	NFI	RMSEA	SRMR
Four-factor model (MO)	559.315	275	0.939	0.956	0.957	0.887	0.058	0.0509
Three-factor model (M1)	155.587	67	0.941	0.956	0.959	0.963	0.061	0.0356
Two-factor model (M2)	62.714	35	0.967	0.971	0.981	0.961	0.051	0.0387
One-factor model (M3)	124.885	7	0.825	0.875	0.868	0.885	0.266	0.0567

**Table 6:** Construct validity.

Construct	Number of dimensions	Factor loading	AVE	CR	Cronbach’s alpha
Perceived CSR (PCSR)	PCSR 1	0.71	0.582	0.871	0.817
	PCSR 2	0.87			
	PCSR 3	0.81			
	PCSR 4	0.73			
	PCSR 5	0.85			
	PCSR 6	0.71			
Ethical leadership (EL)	EL 1	0.73	0.644	0.853	0.875
	EL 2	0.75			
	EL 3	0.82			
	EL 4	0.83			
	EL 5	0.76			
	EL 6	0.75			
	EL 7	0.71			
	EL 8	0.86			
	EL 9	0.85			
	EL 10	0.87			
Moral reflectiveness (MR)	MR 1	0.89	0.675	0.825	0.851
	MR 2	0.88			
	MR 3	0.75			
	MR 4	0.87			
	MR 5	0.71			
Pro-environment behavior (PEB)	PEB 1	0.61	0.531	0.921	0.915
	PEB 2	0.69			
	PEB 3	0.76			
	PEB 4	0.78			
	PEB 5	0.81			
	PEB 6	0.66			
	PEB 7	0.74			
	PEB 8	0.78			
	PEB 9	0.76			
	PEB 10	0.83			
	PEB 11	0.85			
	PEB 12	0.87			

**Table 7:** Discriminant validity.

Constructs	CR	AVE	MSV	MaxR(H)	PCSR	PEB	MR	EL
PCSR	0.878	0.581	0.381	0.910	0.771			
PEB	0.921	0.532	0.459	0.919	0.533	0.686		
MR	0.824	0.671	0.493	0.861	0.618	0.677	0.810	
EL	0.851	0.641	0.491	0.895	0.512	0.669	0.710	0.791

CSR, corporate social responsibility; MR, Moral reflectiveness; EL, ethical leadership; PEB, pro-environmental behavior; CR, Composite Reliability; AVE, average variance extracted; MSV, maximum shared variance; MaxR(H), McDonald Construct Reliability.

### Structural model

The proposed model was evaluated to several satisfactory goodness of fit metrics based on values recommended by Hair et al. (2010) and Hu and Bentler (1999). [*χ*^2^ = 228.981, *df* = 112, *χ*^2^/*df* = 1.959, Root Mean Square Error of Approximation (RMSEA) = 0.062, Goodness of Fit Index (GFI) = 0.926, Adjusted Goodness of Fit Indices (AGFI) = 0.889, Normed Fit Index (NFI) = 0.937, Routine Fit Index (RFI) = 0.915, Incremental Fit Index (IFI) = 0.970, Tucker–Lewis Index (TLI) = 0.960, and Comparative Fit Index (CFI) = 0.970]. H1, H2, H3, H4, and H5 were supported and statistically significant impacts of perceived CSR on pro-environmental behavior, Moral reflectiveness, and ethical leadership (see Table 8). Furthermore, the conceptual model indicated that two mediators: Moral reflectiveness; and ethical leadership, would have a significant and positive impact on employees’ pro-environmental actions. Table 6, shows the standardized estimates and their 95 percent confidence intervals, which were calculated using 5,000 bootstrapped samples (Jose, 2013). The results of standardized estimates of indirect values showed that Moral reflectiveness mediated significant mediation between perceived CSR and employees’ pro-environmental behavior, proving H7. Furthermore, the second mediating association of ethical leadership was shown to be statistically significant, confirming H6 (Table 9).

**Table 8:** Regression results of the structural model and hypotheses test outcomes.

**Hypothesis**	**Predicted relationship**	**Standard path loadings**	**Standard error**	***t*-value**	**value of *p***	**Decision**
H1	Perceived CSR → PEB	0.55	0.80	6.74	0.002	Supported
H2	Perceived CSR → EL	0.52	0.85	6.774	0.001	Supported
H3	Perceived CSR → MR	0.71	0.08	7.587	0.003	Supported
H4	EL → PEB	0.55	0.64	6.698	0.003	Supported
H5	MR → PEB	0.52	0.07	6.852	0.001	Supported

CSR, corporate social responsibility; MR, Moral reflectiveness; EL, ethical leadership; PEB, pro-environmental behavior.

**Table 9:** Standardized mediation effects: parameter estimate and bootstrap percentile method confidence intervals.

Hypothesis	Parameter	Estimate	Lower bound	Upper bound	value of *p*	Decision
H6	Panel Ia Perceived CSR → EL → PEB	0.341	0.261	0.430	0.012	Supported
H7	Panel Ib Perceived CSR → MR → PEB	0.271	0.183	0.358	0.010	Supported

CSR, corporate social responsibility; MR, Moral reflectiveness; EL, ethical leadership; PEB, pro-environmental behavior.

^a^Goodness of fit: *χ*^2^/df = 1.958, RMSEA = 0.051, GFI = 0.925, CFI = 0.957 (“Perceived CSR → MR” was constrained to be zero).

^b^Goodness of fit: *χ*^2^/df = 2.325, RMSEA = 0.055, GFI = 0.925, CFI = 0.962 (“Perceived CSR → EL” was constrained to be zero).

## Discussion

This research study examined into how perceived CSR effects pro-environmental behavior through the employees’ moral reflectiveness and ethical behavior. The results suggest that PCSR positively and directly effects pro-environmental behavior, employees’ moral reflectiveness, and ethical leadership. Most past studies have investigated the impact of perceived CSR on employee behavioral outcomes and attitudes, but few studies have been conducted in the SME industry of Pakistan (Wu et al., 2021). Furthermore, this study provided evidence for the empirical association among the variables in SME industry of Pakistan. This study emphasizes on the importance of pro-environmental behavior in SMEs and its role in the country GDP as it is human intense sector. The findings of this study show that PCSR positively and directly effects pro-environmental behavior, employees’ moral reflectiveness, and ethical leadership. More specifically, the findings show that employees with a high moral reflectiveness exhibit more environmentally responsible behavior. Interestingly, the findings also show that PCSR through ethical leadership enhances the pro-environmental behavior. According to the findings. By elucidating the sociopsychological dynamics that underpin the relationship between perceived CSR and pro-environmental workplace behavior, the study revealed that employee’ perceptions of CSR can influence their pro-environmental behavior both directly and indirectly *via* moral reflectiveness and ethical leadership. This is consistent with prior research, which has found both a direct and indirect effect of CSR perceptions on employee pro-environmental behavior in a variety of contexts and organizational settings (Babiak and Trendafilova, 2011; Gkorezis and Petridou, 2017; Afsar et al., 2018; Islam et al., 2019). The findings of this study further extend the work of Afsar and Umrani (2020), where there was a direct and indirect link between employee pro-environmental behavior and perceived CSR was suggested. Moreover, this study employed the AMO theory to examine the impact of CSR, EL on PEB. The results of this study are consistent with the AMO theory which reflect the notion that employee’s behavior and their valuable environmental ideas which are unique and combined at individual and situational elements promoted through CSR and EL (Jia et al., 2018; Sidney et al., 2022).

This study findings further strengthen the notion that ethical leadership enhance the pro-environmental behavior and found to be the key driver to trigger the pro-environmental behavior. This further opens new avenue for the organization to benefit by inculcating the pro-environmental behavior through perceived CSR practices and the ethical leadership role. Perceived CSR and ethical leaders can foster the employees’ sustainable values and trigger their priorities toward environment. In SMEs, the middle-line manager’s perceived CSR practices are important in a way that it affects the ethical leadership which, in turn, trigger the environmental behavior. Moreover, in SMEs, there is also a need to establish the culture of pro-environmental practices and green values which pave the way for new employees to adopt the same practices as an organizational culture. The findings of the study have several implications for the strategic management and general management. This study provides insightful evidences in context of developing country like Pakistan. These empirical evidences show the importance of CSR and ethical leadership impact on SMEs which is positive and significant. Further, it provides the evidences that CSR *via* EL can be more effective for SMEs to grow and compete. The objective of this study is to present the empirical evidence that help the practitioners of SMEs to enhance their productivity and PEB along with that to provide the new avenue for researchers who are working in the CSR, leadership and PEB research field. Based on the results, it can be concluded that CSR and EL can lead toward better PEB. Therefore, for practitioners in the SMEs, they need to be of mindful of their CSR practices along with their EL style and should master the EL style and its implementation in the organization to educate and promote the environmental conservation practices. In addition to that, SME practitioners need to introduce the proper CSR practices and EL which promote the sense of responsibility in employees toward the environment in today’s challenging world of globalization. Moreover, in order to be successful SME, the management should be competent enough to channelize the CSR and EL in a most effective way to use these as a triggers and drivers to influence the employee’s behavior toward pro-environment while making the different decisions. Further, SMEs need to build and promote the CSR and EL as their mantra to take their firms forward. This is also supported by the theory of AMO and the components of AMO which further strengthen the employee’s attachment and their responsibility with the work environment.

The findings suggest that, in addition to directly improving pro-environmental behavior by CSR perception, it is further strengthened through Moral reflectiveness. This entails the investigation for mediation mechanisms between the perceived CSR and pro-environmental behavior and opens the way for more research in the future. By combining the two mediators (Moral reflectiveness and ethical leadership), at the same time in a single model, the suggested model adds to previous pro-environmental behavior models that were limited to direct linkages or simple mediation (Afsar and Umrani, 2020).

### Theoretical implications

The study’s findings add to the existing literature on CSR and environmental management by providing useful theoretical insights. First, it adds to prior studies on employee pro-environmental behavior by elucidating its antecedents. The findings support the theory that personal attitudes influence behavior (moral reflectiveness) and ethical leadership and integrate in a coherent and interconnected conceptual framework to influence employee pro-environmental behavior (Afsar and Umrani, 2020). Second, by shedding light on the importance of individual differences in personality characteristics and moral reflectiveness, our research contributes to a growing understanding of environmental sustainability in the context of SME sector. Moral reflectiveness may be a proximal driver of pro-environmental conduct at work for employees. In the domestic and in social settings, personal moral norms play an essential influence in pro-environmental conduct (Gärling et al., 2003; Bamberg and Möser, 2007). Our analysis provides another theoretical route for inducing employee pro-environmental behavior by identifying generic personality and moral features that may stimulate employees’ underlying motives for pro-environmental activities. Third, the study extends the findings of Afsar and Umrani (2020), which reported that moral reflectiveness to mediate between CSR and PEB and suggested to add more mediator to explore further the effect of CSSR on PEB.

### Managerial implications

This study shows that employees’ perceptions of their company’s involvement in social responsibility motivate them to engage in pro-environmental activities. Organizations may hold meetings, workshops, or seminars to raise awareness about their CSR efforts. As a result, if SMEs want to encourage environmental behavior in the workplace, they may take an advantage from implementing CSR practices on a personal level. According to our findings and empirical evidences, moral reflectiveness may have a role in employees’ pro-environmental conduct (Van Velsor and Quinn, 2012; Afsar and Umrani, 2020). It is crucial to put in place that firms should keep their employees informed about their efforts, CSR projects and activities, and environmental policies on a regular basis (Rupp et al., 2013). Employees are sometimes unaware of firms’ solutions and ways for addressing social responsibility issues. Employees can regard CSR initiatives with pessimism and mistrust owing to a lack of communication, even when firms really invest and genuinely try to benefit society and the environment (Wry et al., 2006; Afsar and Umrani, 2020). In addition to that employees’ worries, uncertainties, and fears concerning environmental management and social responsibility efforts should also be addressed by management. In this aspect, actual corporate greening is required rather than symbolic greening (Boiral, 2007; Collier and Esteban, 2007), if the goal is to develop widespread employee support for environmental improvement.

### Limitations and future research directions

There are several limitations to this study that should be mentioned. Despite the fact that this study carefully picked a pro-environmental behavior measure that assesses how employees go about initiating rather general and unspecific environmental actions that may be used to a variety of situations, “organizations, activity sectors, occupations or circumstances” (Boiral and Paillé, 2012, p. 435), Future research should look at how individual and organizational characteristics influence employee engagement in environmental issues within the same industry sector or company, focusing on individuals who are more typical of the entire workforce. This research focused on organizations primarily based in Punjab, future research should gather data from other provinces to have a better representation of SMEs. Furthermore, in order to improve future research findings, comprehensive measures of moral reflectiveness are required. Moreover, the nature of this study, which is cross-sectional, in future longitudinal studies should be conducted to gain more insights about all these relationships. In addition to that, future studies should consider the SME size for implications or to investigate the role of PCSR and ethical leadership in bigger and small SMEs and compare their results for more in-depth analysis.

## Data availability statement

The raw data supporting the conclusions of this article will be made available by the authors, without undue reservation. Requests to access these datasets should be directed to haidershah11@gmail.com.

## Author contributions

SS and MM worked on overall paper introduction and literature review and developing the conceptual framework. NA worked on the paper methodology and data analysis. BA-G worked on conclusion and implications of the study. All authors contributed to the article and approved the submitted version.

## Conflict of interest

The authors declare that the research was conducted in the absence of any commercial or financial relationships that could be construed as a potential conflict of interest.

## Publisher’s note

All claims expressed in this article are solely those of the authors and do not necessarily represent those of their affiliated organizations, or those of the publisher, the editors and the reviewers. Any product that may be evaluated in this article, or claim that may be made by its manufacturer, is not guaranteed or endorsed by the publisher.
